# Single dose of Glycoprotein K (gK)-deleted HSV-1 live-attenuated virus protects mice against lethal vaginal challenge with HSV-1 and HSV-2 and induces lasting T cell memory immune responses

**DOI:** 10.1186/1743-422X-10-317

**Published:** 2013-10-28

**Authors:** Arun V Iyer, Bapi Pahar, Vladimir N Chouljenko, Jason D Walker, Brent Stanfield, Konstantin G Kousoulas

**Affiliations:** 1 Division of Biotechnology and Molecular Medicine, and Department of Pathobiological Sciences, Louisiana State University School of Veterinary Medicine, Skip Bertman Drive, Baton Rouge, LA 70803, USA; 2 Division of Comparative Pathology, Tulane National Primate Research Center, 18703 Three Rivers Road, Covington, LA 70433, USA; 3 Current Address: Boehringer Ingelheim Vetmedica, 2501 North Loop Dr, Suite 1000, Ames, IA 50010, USA

## Abstract

**Background:**

Herpes simplex virus type-1(HSV-1) and HSV-2 are important human pathogens that cause significant ocular and urogenital complications, respectively. We have previously shown that HSV-1 virions lacking glycoprotein K (gK) are unable to enter into neurons via synaptic axonal membranes and be transported in either retrograde or anterograde manner. Here, we tested the ability of HSV-1 (F) gK-null to protect against lethal challenge with either highly virulent ocular HSV-1 (McKrae strain), or genital HSV-2 (G strain). The gK-null virus vaccine efficiently protected mice against lethal vaginal infection with either HSV-1(McKrae) or HSV-2 (G).

**Results:**

Female mice were immunized via a single intramuscular injection with 10^6^ PFU of the gK-null virus. Immunized mice were treated with Depo-Provera fourteen days after vaccination and were challenged via the vaginal route one week later. Ninety percent of mice vaccinated with the gK-null virus survived HSV-1 (McKrae) challenge, while 70% of these mice survived after HSV-2 (G) challenge. Moreover, all vaccinated mice exhibited substantially reduced disease symptoms irrespective of HSV-1 or HSV-2 challenge as compared to the mock vaccinated challenge group. T-cell memory immune responses to specific glycoprotein B (gB) and glycoprotein D (gD) peptide epitopes were detectable at 7 months post vaccination.

**Conclusions:**

These results suggest that the highly attenuated, non-neurotropic gK-null virus may be used as an effective vaccine to protect against both virulent HSV-1 and HSV-2 genital infections and induce lasting immune responses.

## Introduction

HSV-1 and HSV-2 are closely related viruses sharing 83% nucleotide homology [1]. However, these viruses cause different spectrum of disease symptoms. HSV-1 is the causative agent of cold sores, herpetic whitlow, herpes keratitis and ocular infections [2]. It is also a leading cause of infectious blindness in the United States [3]. HSV-2 however, is primarily a sexually transmitted disease more often restricted to the genitals [4], although in recent years, HSV-1 has been increasingly identified with genital herpes infections [5-7]. Both HSV-1 and HSV-2 produce persistent lifelong infections by establishing latency in immune privileged sensory neurons [8]. Herpes infections can carry significant social implications, and the economic costs associated with genital herpes is substantial (projected to be around $2.5 billion in 2015 and around $3 billion in 2025) [9].

A number of vaccine approaches and candidates have been evaluated in laboratory animals and humans, including purified peptides, recombinant glycoprotein subunits, inactivated, live attenuated, replication competent and replication defective whole viruses among others (reviewed in: [10-13]). Recently, a clinical trial using the gD subunit vaccine produced by GlaxoSmithKline showed that it was effective in preventing HSV-1 genital disease and infection, but not in preventing HSV-2 disease or infection [14].

Replication competent live attenuated virus vaccines have the distinct advantage of replicating in the host’s cells providing a broader spectrum of viral antigens and pathogen associated molecular patterns than non-replicating vaccine approaches. Ideally, a single live attenuated HSV-1 vaccine virus should provide immunogenic determinants shared by both HSV-1 and 2, conferring cross-protection. In addition to being attenuated, such a vaccine should have a high safety profile and not persist i.e., enter latency. A number of herpes viruses with a variety of genetic deletions have been evaluated as live attenuated vaccines. These include deletions in glycoprotein E (gE) [15,16], multiple deletions in γ34.5, UL55-56, UL43.5, US10-12 [17], UL5, UL29, UL42, ICP27 genes [18-21], deletion of ICP0^-^[22] and the UL9 genes [23-26].

In this paper, we describe the use of a glycoprotein K-deleted HSV-1 (gK-null) virus as an effective vaccine to protect against lethal challenge with HSV-1 and HSV-2. We have previously shown that gK plays a significant role in HSV-1 infection of cells and is vital to the cellular spread of HSV-1 [27-34]. Previous research from our laboratory indicates that HSV-1 mutants lacking gK are unable to efficiently infect and establish latency in neurons [3]. Apparently, this stems from the inability of gK-null virions to efficiently infect neurons via synaptic axonal membranes [35]. As a result, this attenuated virus is an ideal vaccine candidate; it is safe, while at the same time it is capable of presenting, with the exception of gK, the full spectrum of viral antigens and innate immune triggers. Additionally, gK has been implicated as a viral glycoprotein that enhances virulence and exacerbates acute ocular disease [36-39], and its absence is likely to enhance safety.

Here, we report that a single inoculation with the gK-null virus successfully protects mice against lethal vaginal challenge with the highly neurovirulent HSV-1 (McKrae), or HSV-2 (G), reducing both the severity and occurrence of disease symptoms. The gK-null vaccine protected 90% of the mice against HSV-1 and 70% of the mice against HSV-2 lethal infection. Analysis of T-cell responses showed that the vaccine produced long-term memory T cell responses to known CD4+ and CD8+ epitopes of HSV-1 glycoproteins gB and gD.

## Results

### HSV-1 and HSV-2 challenge following vaccination with gK-null

**Figure 1:**
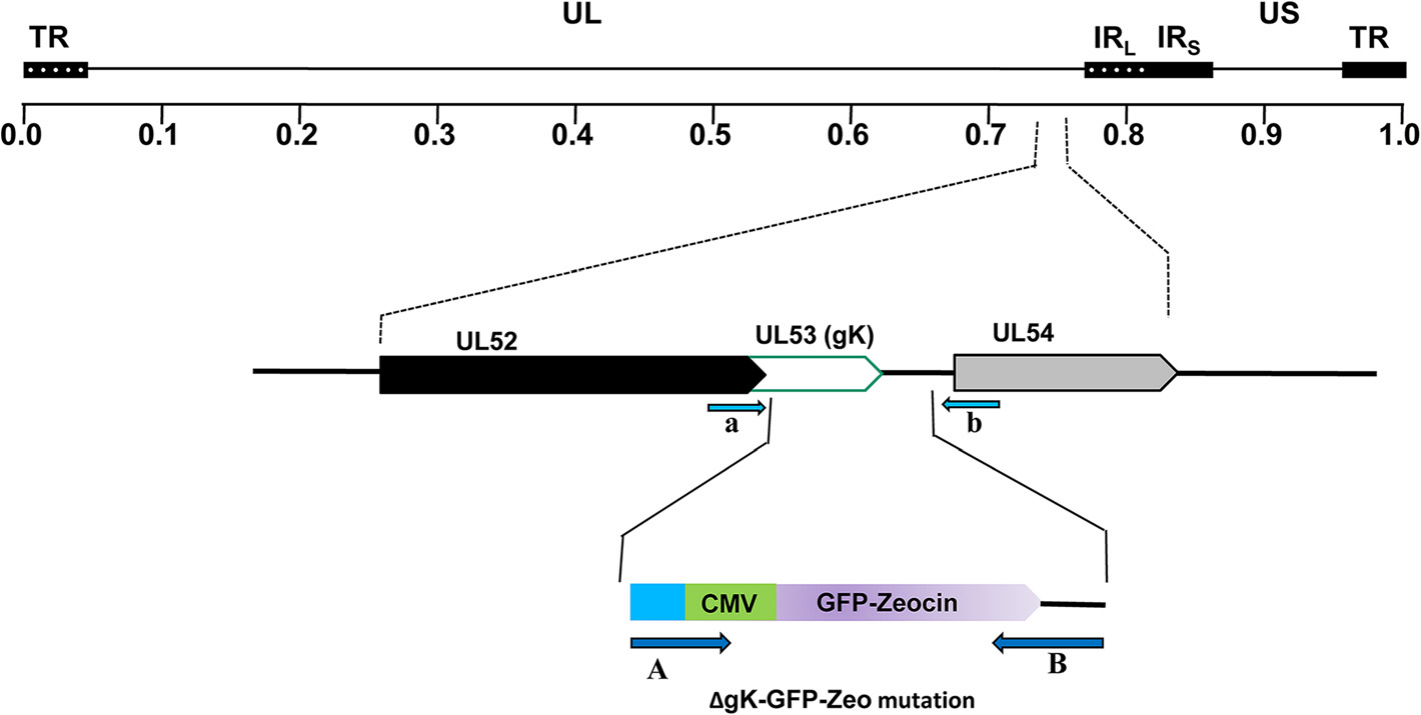
**Schematic of the strategy for the construction of pYEbac102 mutant BAC.** The top line represents the prototypic arrangement of the HSV-1 genome, with the unique long (UL) and unique short (US) regions flanked by the terminal repeat (TR) and internal repeat (IR) regions. Shown below are the expanded genomic regions of the UL53 ORFs as well as the approximate locations of the sites to which insertion of the marker genes was targeted and the primers used in diagnostic PCR to confirm the presence of each mutation. PCR fragments containing the GFP-zeocin resistance gene cassette flanked by 50 bp of viral sequences on both sides were used for targeted recombination in *E. coli* to construct pYEbac102 mutant BAC with insertion-deletion mutations in the UL53 ORF. The approximate locations of the primers used in amplification of each PCR fragment are also shown.

**Figure 2:**
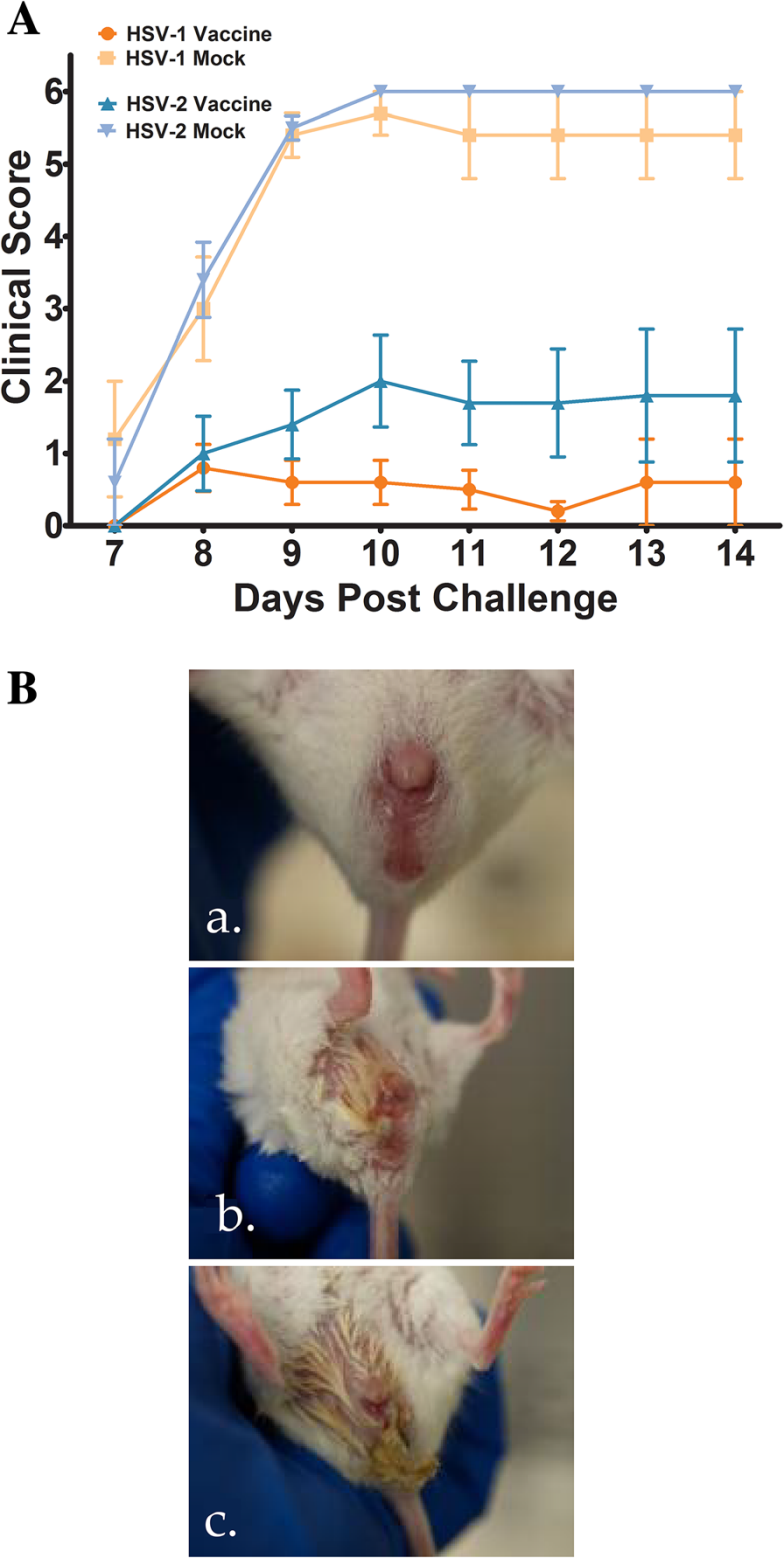
**Clinical disease score and pathology. (A)** Clinical disease scores for vaccinated and mock vaccinated animals challenged with HSV-1 (McKrae) or HSV-2(G). Detailed clinical scores from seven days post challenge was recorded. Scores on a scale of 1–6 were assigned based on the following scheme: 0 = no disease, 1 = ruffled fur and generalized morbidity, 2 = mild genital erythema and edema, 3 = moderate genital inflammation, 4 = genital inflammation with purulent discharge, 5 = hind limb paralysis and 6 = death. Animals showing hind limb paralysis were immediately euthanized and scored as dead from the following day. In both HSV-1 (McKrae) and HSV-2(G) challenge groups, a significant difference was observed between mock vaccinated and vaccinated animals (p = 0.0005) using a two-tailed paired T-test. **(B)** HSV-1(McKrae) and HSV-2(G) infected mice exhibited similar disease progression and pathology. **(a)** Mock-infected vagina. **(b)** Mild disease symptoms included ruffled fur, hunching posture, inflammation and redness of vagina. **(c)** More serious manifestations included purulent vaginal discharge.

The gK-null virus was constructed by deletion of the gK gene using double-red recombination in conjunction with the HSV-1 genome cloned into a bacterial artificial chromosome (see Materials and Methods) (Figure 1). We have previously shown that this HSV-1 F gK-null virus has reduced replication ability in non-complementing Vero cells [40]. Upon vaccination, mice were observed daily for clinical symptoms and adverse reactions to the vaccine. Vaccinated animals showed no apparent injection site reactions (not shown) nor did they present with any disease symptoms prior to challenge. Following vaginal challenge with HSV-1 and HSV-2 all animals were observed on a daily basis for disease manifestation. Clinical scores and observations were recorded for 14 days post-virus challenge (Figure 2A). Mock-vaccinated animals challenged with either HSV-1or HSV-2 exhibited pronounced clinical signs to various degrees. Disease symptoms in the mildest cases consisted primarily of hair loss, hunched posture and fur ruffling. More advanced disease symptoms included vaginal and peri-anal erythema and edema, accompanied by purulent discharge (Figure 2B). The manifestation of symptoms was similar between HSV-1 and HSV-2 challenged mice within each group (not shown).

**Figure 3:**
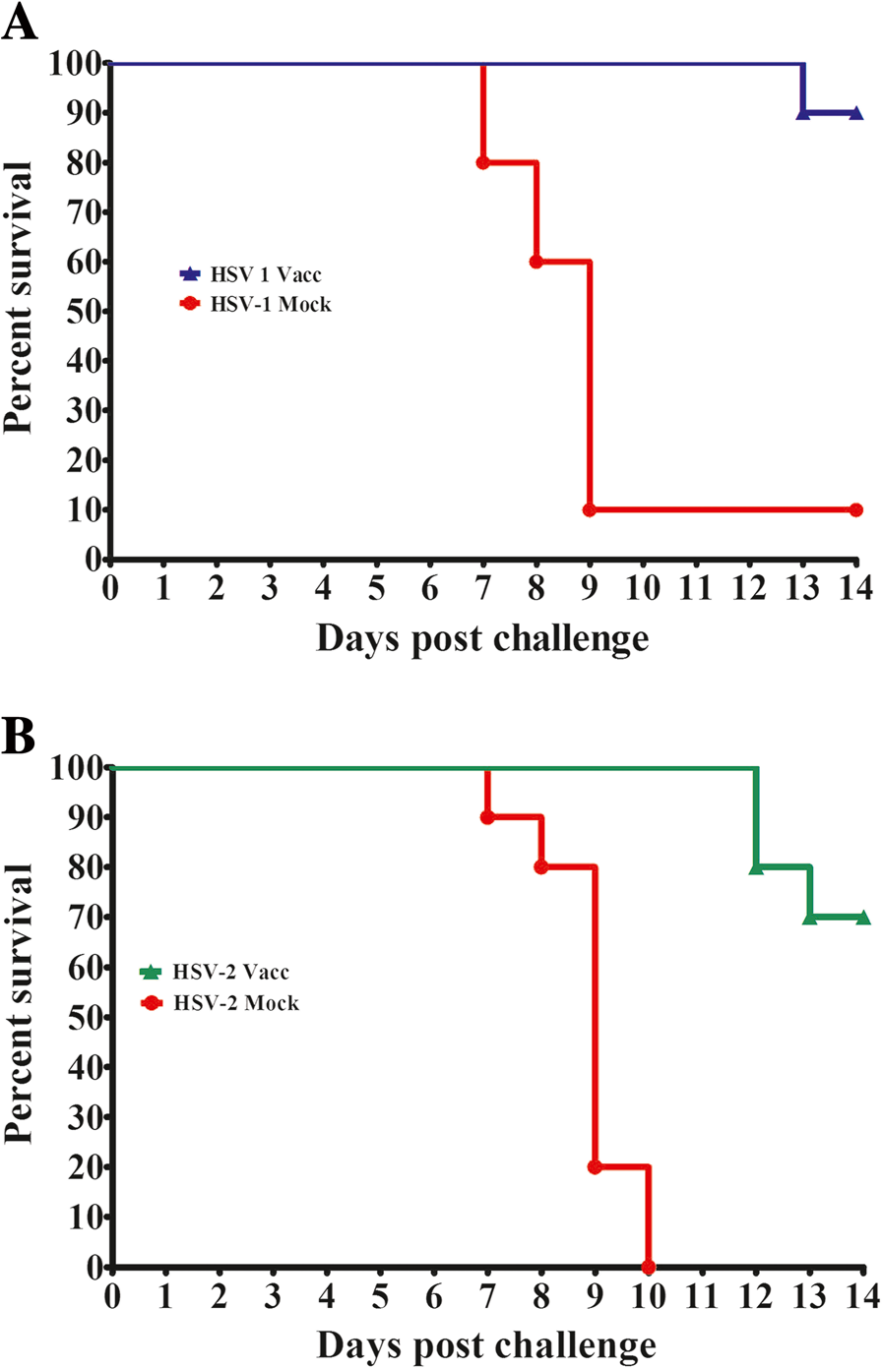
**Kaplan-Meier survival curves.** Vaccinated and mock-vaccinated mice were challenged thorough the intra-vaginal route with 10^6^ PFU of HSV-1 McKrae **(A)** or HSV-2(G) **(B)** 21 days post primary vaccination and observed for 14 days. Ninety percent of the vaccinated animals in the HSV-1 challenged group survived, while 90% of the mock-vaccinated animals died **(A)**. A statistically significant difference was observed between the vaccinated and mock-vaccinated groups (*p* = 0.0002) using the Gehan-Breslow-Wilcoxin test. Among animals challenged with HSV-2(G), 70% of the vaccinated group survived while 100% of the mock-vaccinated animals died **(B)**. A statistically significant difference was observed between the vaccinated and mock-vaccinated groups (*p* = 0.0001) using the Gehan-Breslow-Wilcoxin test.

Mortality was delayed in the vaccinated groups. Within both the HSV-1 and HSV-2 challenge groups, mock vaccinated animals started to die as early as 7 days post challenge with a majority of deaths occurring on day 9. In contrast only a single animal died on day 13 in the vaccinated groups challenged with HSV-1. Similarly, deaths among vaccinated animals challenged with HSV-2 occurred on days 12 and 13. Protection against lethal infection was significantly higher in the vaccinated group compared with the control for HSV-1 challenge: 90% vs. 10% (*p* = 0.0002) (Figure 3A). Moreover vaccinated animals showed a reduction in the severity and occurrence of disease symptoms post-challenge when compared with controls. We observed similar results in the HSV-2 challenge groups. Seventy percent of the vaccinated animals survived intra-vaginal challenge with HSV-2, while none of the mock-vaccinated animals survived the challenge (*p* = 0.0001). The majority of deaths occurred on day 9 in the mock group compared with day 12 in the three vaccinated mice that died after HSV-2 challenge (Figure 3B). Recently, we have performed similar experiments with new gK-null-like mutant viruses that contain portions of gK and have obtained similar clinical symptoms and protection results (data not shown; Stanfield et. al., manuscript in preparation).

### T cell memory responses to HSV-1 targets in vaccinated animals

**Figure 4:**
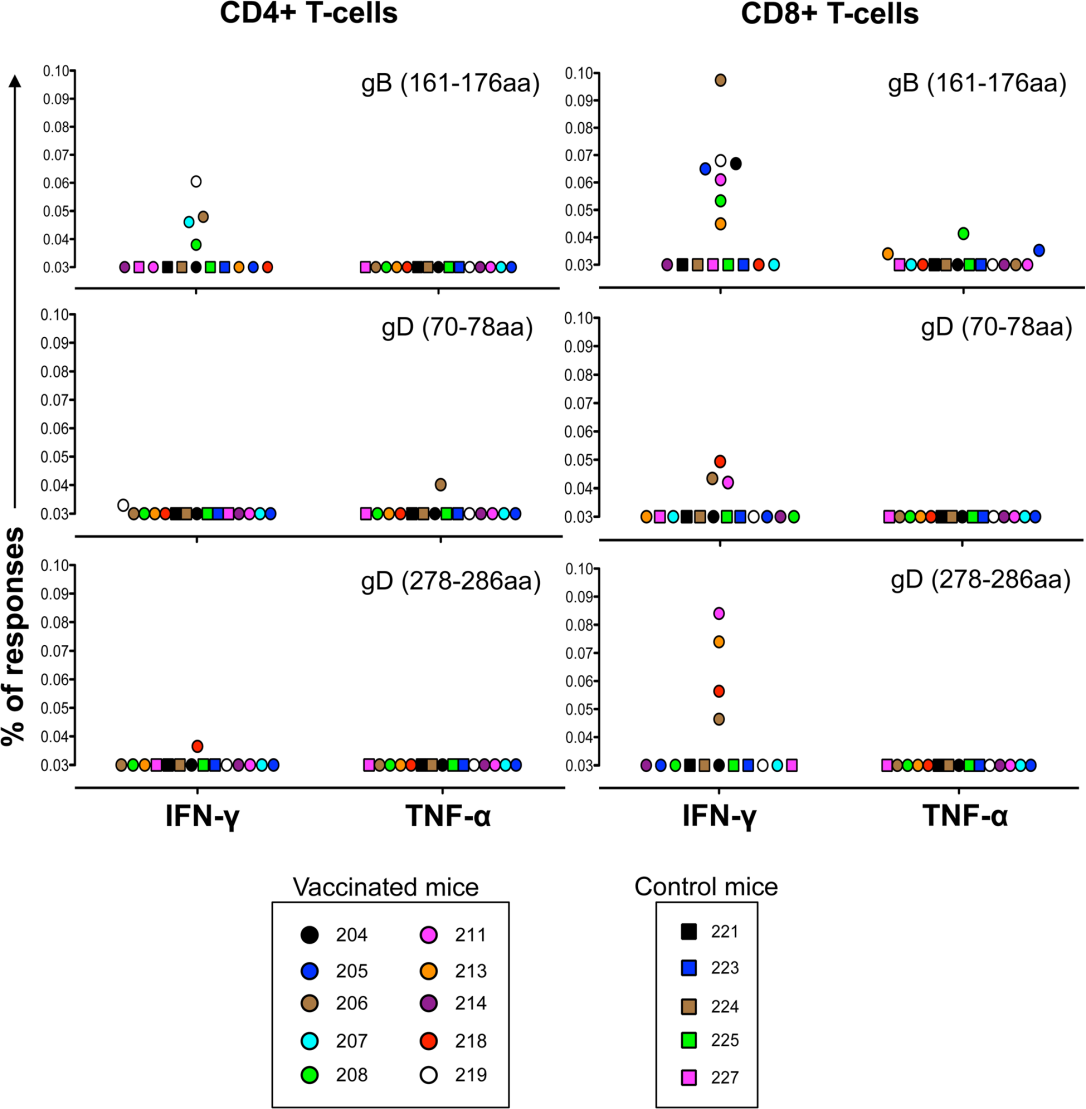
**CD4+ and CD8+ T-cell responses to HSV-1 vaccine antigens.** Cells were stimulated with either HSV-1 gB or HSV-1 gD peptide ex vivo for 6 hrs and then stained for the detection of antigen-specific IFN-γ and TNF-α cytokine responses. The responses for each mouse for the specified antigen are shown in scattered plots. The cutoff value for IFN-γ and TNF-α cytokine responses were based on the data generated from the mock control mice. Percentages of responses greater than 0.03 are considered positive for the specified antigen and cytokine response. Media control values were subtracted from each value before the analysis.

To assess the establishment of T cell memory directed against HSV-1 epitopes, splenocytes from vaccinated and control mice were collected 7 months post vaccination and stimulated with purified peptide fragments derived from major HSV-1 surface antigens gB and gD. Following ex vivo peptide stimulation, the splenocytes were assessed for activation of CD4+ and CD8+ T cells using intracellular IFN-γ and TNF-α as markers of activation. Representative results and gating strategies from flow cytometry are -shown in Additional file 1: Figure S1. Peptide stimulated splenocytes from vaccinated mice produced IFN-γ and/or TNF-α positive T cell frequencies above a baseline established using mock vaccinated mice. IFN-γ responses were more common in both CD4+ and CD8+ T cell populations than TNF-α. T cell responses to the gB (161–176) peptide were most common among the epitopes tested, and, CD8+ T cell responses were more frequently observed than CD4+ (Figure 4) with 70% producing antigen specific CD8+ IFN-γ in response to gB (161–176) peptide (Figure 4 top right panel).

### T cell phenotypes in isolated splenocytes

**Figure 5:**
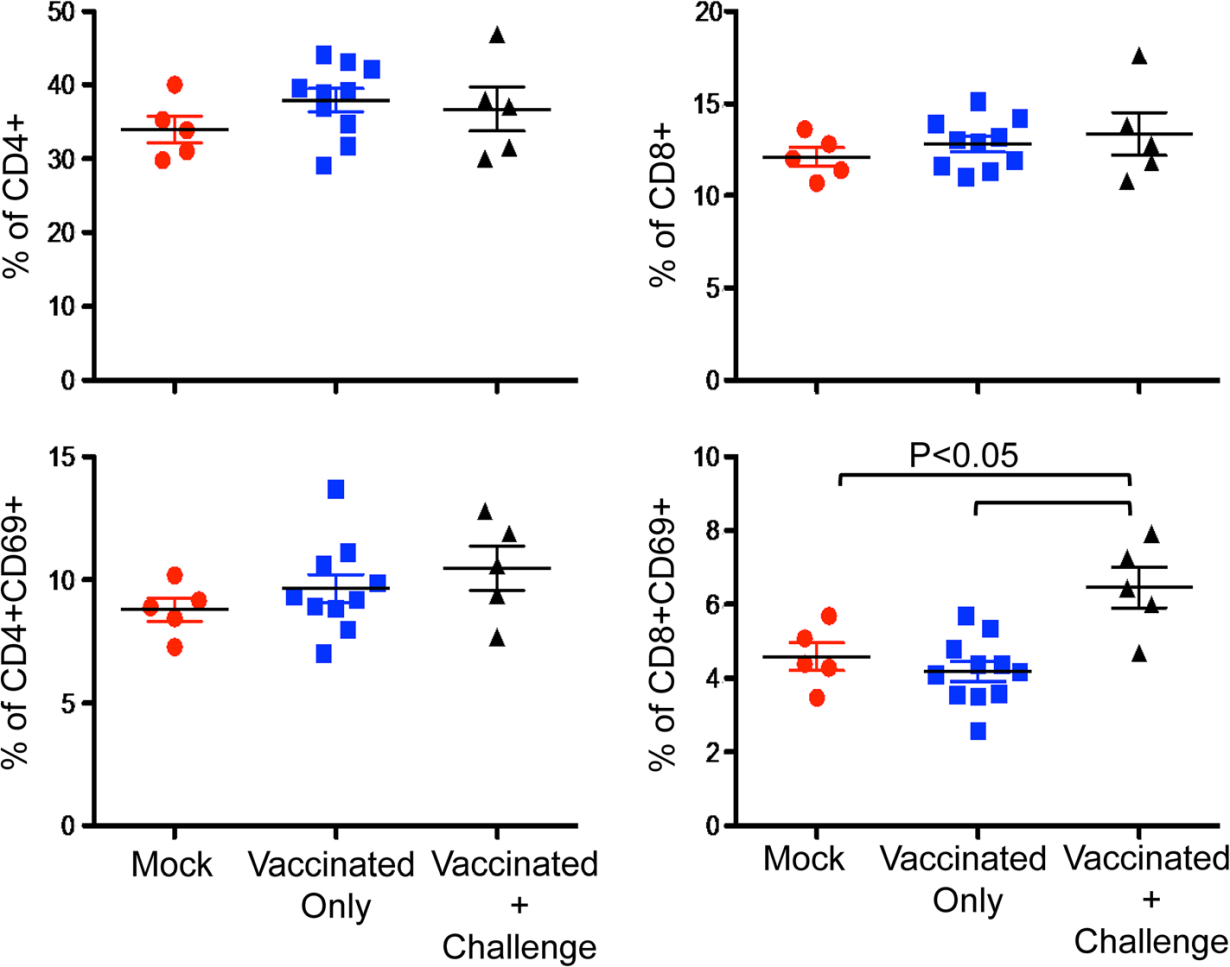
**CD4 and CD8 T lymphocyte counts and their activation in mock, vaccinated and vaccinated-challenged mice.** No substantial changes in CD4 and CD8 population were noticed within mock, vaccinated and vaccinated-challenged group of mice. However, there was an increased CD8 + CD69+ T cell activation monitored in vaccinated-challenged mice compared to other groups (P < 0.05).

The overall percentages of CD4 and CD8 T cells in isolated splenocytes, without ex vivo activation, were determined for all unvaccinated, HSV-1 vaccinated and HSV-1 vaccinated-challenged mice. There were no significant differences in CD4+ and CD8+ T cell population percentages among all the groups of mice tested (Figure 5, upper row). CD69 surface expression was used as an early activation marker of T cells to indicate activation status [41]. The mean percentages of CD69 positive CD4+ T cells were 9.6% in vaccinated mice and 8.8% in mock vaccinated mice and were not significantly different. In contrast, a significantly higher proportion of splenic CD8+ T cells were positive for CD69 in the vaccinated and challenged group of animals when compared to other groups of mice (p < 0.05) (Figure 5, bottom row).

### Profile of regulatory T cell population in vaccinated versus mock-vaccinated mice

**Figure 6:**
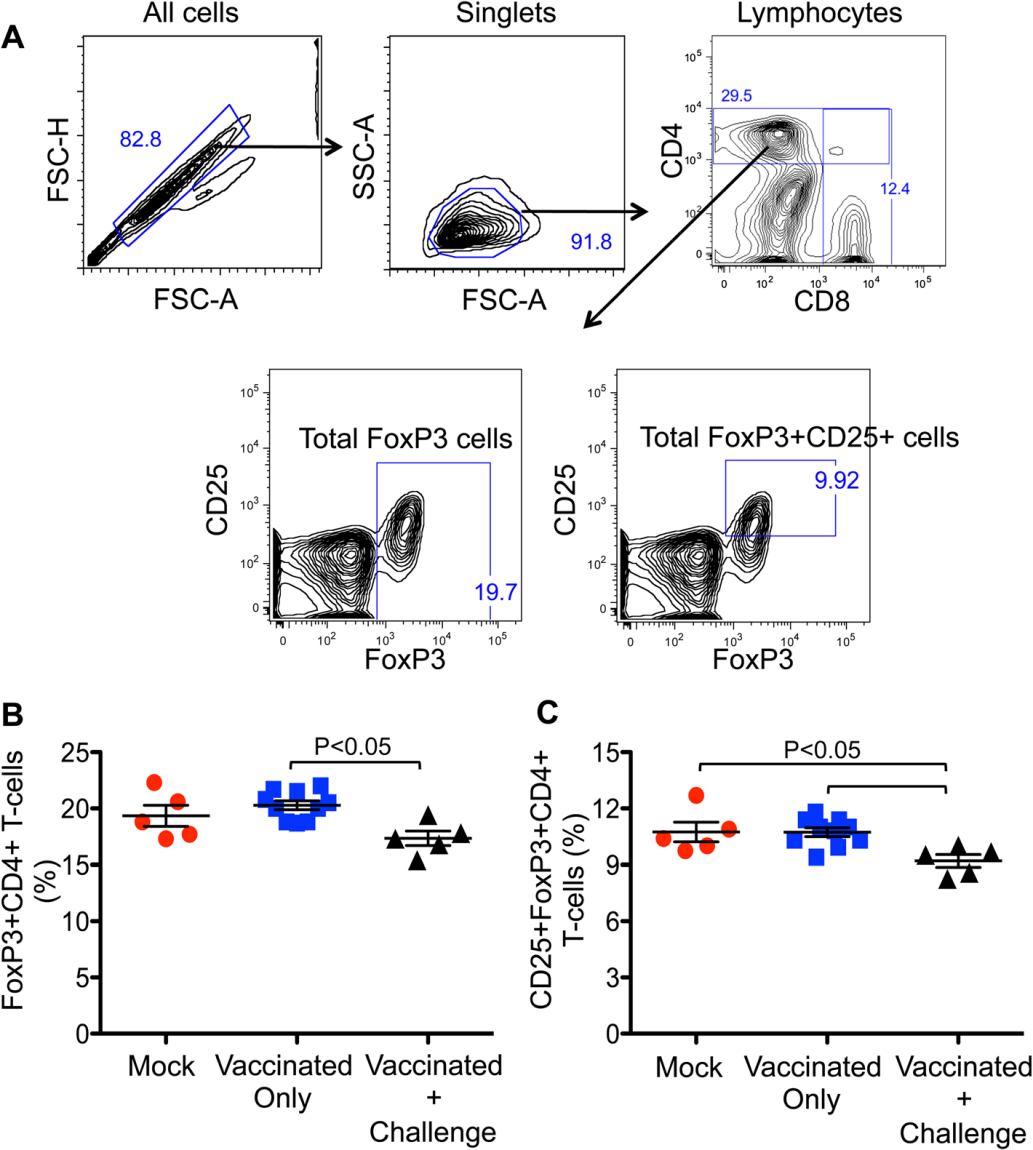
**Role of regulatory T cells in mock, HSV-vaccinated and HSV-vaccinated-challenged mice. (A)** Representative contour plots showing the gating strategy for total FoxP3 and FoxP3 + CD25+ CD4+ Treg cells from splenocytes of a mock vaccinated mouse. **(B & C)** Percentages of total FoxP3 and CD25 + FoxP3+ CD4+ T-reg cells in three different groups of mice. A statistically significant difference (P < 0.05) was observed between HSV-vaccinated- and challenged as compared to vaccinated mice that were not challenged.

FoxP3 is a classic and specific marker expressed in all CD4+ Treg cells that have regulatory activity. The combination of the three markers CD4+, CD25+, FoxP3+ reveals the presence of activated Tregs that typically dampen the immune response. Reduction of Tregs correlates with increased vaccine efficacy. We examined both total FoxP3+ Treg cells, and CD4 + CD25 + FoxP3+ Treg cells to assess their dynamics in all mock and vaccinated groups of mice (see Figure 6A for gating strategy). Total FoxP3 expression in CD4+ T cells from vaccinated mice compared to the mock-vaccinated mice, was not significantly different. Similarly, the CD4 + CD25 + FoxP3+ Treg populations were not different between mock and HSV- 1 vaccinated mice (Figure 6B). However, FoxP3+ cells and CD4 + CD25 + FoxP3+ Treg cells were significantly reduced in vaccinated-challenged mice compared to vaccinated only mice (Figure 6).

## Discussion

We have previously shown that HSV-1 virions lacking glycoprotein K (gK) are unable to infect neurons and establish latency [3]. Moreover, gK-null virions are unable to be transported in either retrograde or anterograde directions in neuronal cell cultures [35]. Here, we show that a single inoculation of the replication competent HSV-1 (gK-null) was sufficient to provide a substantial and significant degree of protection against both virulent HSV-1 and HSV-2 strains in a murine vaginal challenge model. Vaccination caused persistent HSV-1/HSV-2-specific CD4+ and CD8+ T-cell mediated immune responses to essential viral antigens, which were further enhanced upon challenge with either HSV-1 or HSV-2 viruses.

### Vaccination and challenge with HSV-1 and HSV-2

HSV-1(F) is substantially less virulent than HSV-1 (McKrae) via ocular or epithelial routes (unpublished observations); therefore the HSV-1(F) gK-null virus utilized here is predicted to be even more defective for neurovirulence than a HSV-1(McKrae) gK-null virus. This conclusion is supported by the observations that intramuscular injection of mice with the HSV-1(F) gK-null did not cause clinical disease symptoms.

Typically, mice are resistant to either HSV-1 or HSV-2 challenge via the genital route. However, systemic pre-treatment of animals with Depo-Provera has been shown to increase susceptibly of mice to herpes simplex infections, in part because it causes localized immune suppression [42]. In these vaccination-challenge experiments, mice were vaccinated intramuscularly, since this is the most common route utilized by commercially available human vaccines. Vaccinated mice were challenged intra-vaginally with virulent HSV-1 (McKrae) and HSV-2 (G) viruses. Both HSV-1 and HSV-2 strains are commonly found in genital herpes infections, although HSV-1 strains generally appear to cause milder disease symptoms. Surprisingly, HSV-1(McKrae) and HSV-2(G) intra-genital infections at the single dose of 10^6^ PFU produced similar levels of localized inflammation and overall clinical disease symptoms in naïve mice. These results showed that HSV-1(McKrae), originally isolated from a patient with herpes keratitis, remains highly virulent when administered intravaginally in mice, as it has been reported previously [43].

The observed protection from a single dose is significant and subsequent boosts may be expected to enhance survival rates among challenged mice and further reduce disease severity.

To assess whether long-term immunity was established by the HSV-1(gK-null) vaccine, antigen-specific cellular immune responses were determined for selected peptides at approximately 7 months post vaccination. Vaccinated mice exhibited both gB and gD-specific CD4+ and CD8+ T cell mediated immune responses. In relative terms, the most prominent T cell response observed was from gB (161–176) reactive CD8 cells, with 70% of the mice generating IFN-γ positive cells. In human patients, this particular T-cell epitope was identified as recalling the strongest HLA-DR-dependent CD4+ T-cell proliferation and IFN-γ production [44]. Interestingly, these studies identified gB (166–180) and gB (666–680) as a strong CD4+ T cell recall peptides producing cytotoxic T cells that could lyse autologous HSV-1 and gB expressing lymphoblastoid cell lines. These two peptides correlated with asymptomatic (i.e. protective) immune responses suggesting that they may be better candidates in investigating anti-gB CD4+ T cell responses than gB (161–176) utilized in this study [44], provided that these peptides are equally recognized by mouse-specific T-cell responses. The gB (161–176) and gB (166–180) are identical between HSV-1 and HSV-2, therefore, immune responses to these peptides generated by the HSV-1 vaccine could contribute to cross-protection against both HSV-1 and HSV-2 infections. Although the gB (161–176) peptide is known to elicit CD4+ T cell response, it also elicited CD8+ T cell responses in our hands. CD8+ T cell epitope prediction algorithms [45] reveal the potential presence of a CD8+ T cell epitope (TYYKDVTVSQV) within the gB (161–176) peptide. This peptide could be generated after intracellular processing of the gB (161–176) peptide and presented by MHC class I, eliciting CD8+ T cell responses. The two gD peptides used here are HSV-1 specific; however, they differ from HSV-2 gD peptides by only one amino acid each, suggesting that they may also be able to contribute to CD8+ T-cell specific cross-protection against HSV-2 infection.

CD69 is an early T-cell activation marker, which is indicative of recent antigenic stimulation of T cells. Typically, this CD69+ expression subsides as the activated T-cell matures. However, it is known that acute viral infections can produce viral antigen depots in secondary lymphoid organs that can persist for months [46]. Therefore, the observed elevation in CD69+ positive T cells may indicate the presence of either residual HSV-1/HSV-2 antigens, or the presence of reactivated HSV-1/HSV-2 virus that did not cause any appreciable clinical disease symptoms. FoxP3+ reveals the presence of activated Tregs that typically dampen the immune response. Thus, the observed lower levels of these Tregs appear to correlate with enhanced protection against the challenge viruses.

Innate and adaptive immune mechanisms are very important in determining the outcome of an HSV-1/HSV-2 infection. Specifically, production of type I interferon is associated with protection against disease and enhances activation of multiple cell types including natural killer (NK) cells and plasmacytoid dendritic cells (pDCs) thus contributing to anti-HSV immune responses [47]. Adaptive immune responses are thought to play critical roles in disease progression, latency and control of HSV spread [47]. Recently, the importance of cellular immunity was further supported by the prime pull and boost approach in which conventional parenteral vaccination was first utilized to elicit systemic T-cell responses (prime), followed by recruitment of activated T cells by topical chemokine application to the genital tract (pull). This vaccination approach reduced HSV-2 spread into sensory neurons and prevented development of clinical disease [48].

HSV contains several pathogen-associated molecular patterns (PAMPs) that trigger toll-like receptor (TLR) signaling leading to innate immune activation [49]. Subunit and defective herpes virus vaccines are incapable of presenting the complete range of these stimuli which include PAMPs that are only present during late stages of the replication cycle, i.e. double stranded RNA binding to TLR-3. In addition, all herpes viruses cause virus-induced cell fusion as well as fusion of the viral envelope with cellular membranes, which activates innate immunity through the stimulator of interferon genes (STING) [50]. Therefore, a replication competent, but otherwise safe HSV vaccine strain would provide a broader range of immune stimuli not presented via subunit or virus-like particles (VLP) vaccines, which may explain the failure of subunit vaccine to induce satisfactory levels of protection against HSV.

Generally, live-attenuated viral vaccines mimic natural infections and induce more robust humoral and cellular immune responses against a broad spectrum of viral proteins in comparison to subunit vaccines that typically express a single viral protein. Moreover, adaptive immune responses are particularly important in conferring protection against HSV-1 and HSV-2 infections [44,45]. Recently, it was shown that HSV-2-specific CD8+ T cells generated after chemoattractant therapy given vaginally in mice mediated long-lived protection against HSV-2 challenge [46,47]. Our results suggest that immunization with the gK-null virus induces long-term antigen-specific CD8+ T cell responses. It is possible that gK-null immunization induces local intravaginal CD8+ T cell responses that contribute to the observed level of protection against HSV-1 or HSV-2 challenge. Furthermore, gK has been associated with increased virulence and immunopathogenesis. Specifically, ocular infection of mice previously vaccinated with gK exacerbated corneal immunopathogenesis, while a HSV-1 virus expressing two copies of the gK gene was significantly more virulent than the wild-type virus [36,37,48]. Therefore, lack of a functional gK will potentially reduce gK-associated immunopathogenesis, while preventing the virus from infecting ganglionic neurons.

Our results are consistent with the widely accepted notion that pre-existing HSV-1 exposure reduces the severity or duration of HSV-2 acquisition [10]. Ideally, a live-attenuated vaccine could be used for both prophylactic and therapeutic purposes. Elicitation of adaptive immune responses would confer substantial advantage in controlling HSV infections especially, if new ways are found to boost vaccine-induced innate immune activation and subsequent protective adaptive immune responses.

## Materials and methods

### Viruses

HSV-1 (F) gK-null virus has been described previously [40] and was used as the vaccine virus. A schematic for the construction of gK-null is shown in Figure 1. The virus was grown in a gK-complementing VK302 cell line to high titers and passaged once through Vero cells to eliminate carryover of gK containing particles. Cells were freeze-thawed twice to release intracellular viruses and clarified by centrifugation to eliminate cell debris. Virus titers were determined on VK302 and Vero cells. The challenge viruses, HSV-1 (McKrae) and HSV-2 (G) were grown to high titers and titrated in Vero cells. The viruses were assayed for virulence in mice.

### Mouse immunization

All animal studies were carried out after the appropriate approvals were obtained from the Louisiana State University Institutional Animal Care and Use Committee. Six week-old female Balb/c mice (Charles River Laboratories International, Inc., Wilmington, MA, USA) were used in this study. Each mouse was identified with an ear tag (National Band and Tag Company, KY, USA). Mice were divided into two groups to receive either the vaccine or mock inoculations. The animals were mildly anesthetized by inhalation of 2-3% isoflurane and vaccinated intramuscularly with 10^6^ PFU in 100 μl volume of the gK-null virus or with 100 μl of uninfected Vero cell supernatant as sham control.

### Challenge

Fourteen days after primary or mock-vaccination, the animals for challenge were anesthetized by inhalation of 2-3% isoflurane and administered 2 mg Depo Provera (Upjohn, Kalamazoo, MI) subcutaneously under the scruff. Twenty-one days post vaccination (7 days after administration of Depo-Provera) [42], animals in challenge groups (n = 10) were anesthetized with 100 μl xylazine (14.3 mg/ml) and ketamine (1.8 mg/ml) mixture and prepared for challenge. The vaginas of anesthetized animals were swabbed with sterile PBS-soaked Dacron applicators (Puritan, Guildford, ME) to remove associated mucus. Ten microliters (10^6^ PFU) of HSV-1 McKrae or HSV-2G was instilled in the vaginal vault using a micropipette. The mice were held in dorsal recumbency until they recovered from anesthesia. All mice in challenge groups were examined daily for 14 days for signs of disease and death. Animals showing severe neurological symptoms, e.g. ataxia and paralysis, were humanely euthanized.

### Polychromatic flow cytometric staining and analysis

Freshly excised spleens were lightly mashed and mechanically separated using 100 μm nylon mesh cell strainers (Fisher Scientific) in RPMI plus 10% FBS followed by 10 minutes in hypotonic ACK buffer to lyse red blood cells (Invitrogen). Isolated splenocytes were subsequently adjusted to 10^7^ cells/ml. One hundred microliter aliquots of splenocyte suspension were incubated with appropriately diluted concentrations of antibodies for 30 min at room temperature. Cells were washed once with PBS and fixed with 1X BD stabilizing fixative buffer (BD Biosciences) in distilled water. Cells were kept protected from light at 4°C and flow cytometric acquisition was completed within 24 h of staining. Polychromatic (7 parameters) flow cytometric acquisition was performed on a LSR II Becton Dickinson instrument having three lasers (488 nm blue laser, 633 nm red laser and 407 nm violet laser) by using FITC, PE, APC, APC-Cy7 and Pacific Blue as the available fluorochrome parameters. Single-stained controls for each fluorochrome were used for setting flow cytometry compensation. Monoclonal antibodies including anti-CD69 FITC (H1.2F3, BD Biosciences), FoxP3 PE (FJK-16s, eBioscience), CD25 APC (3C7, BD Biosciences), CD4 APC-Cy7 (GK1.5, BD Biosciences) and CD8a Pacific Blue (53–6.7, BD Biosciences) were used. FoxP3 staining was performed by intracellular staining after performing surface staining for all other monoclonal antibodies specified above using the FoxP3 buffer set (Biosciences) and manufacturer’s protocol. At-least 50,000 events were collected by gating on CD4+ T cells and these data were analyzed using FlowJo software version 9.6 (TreeStar Inc.).

To test CD4+ and CD8+ T lymphocyte subsets for IFN-γ and TNF-α production and response to recall antigens, intracellular cytokine flow cytometry assay was employed. Cells were stimulated with either HSV-1 gB (aa 161–176; ATMYYKDVTVSQVWF), gD (aa 70–78; SLPITVYYA) or gD (aa 278–286; ALLEDPVGT) peptides in the presence of 0.5 μg/ml of anti-CD28 (37.51, BD Biosciences) and anti-CD49d (9C10, BD Biosciences) monoclonal antibodies as described previously [51]. Processed splenocytes were resuspended at 10^6^ cells/ml in complete RPMI-10 with 10% FCS, and stimulated with different HSV-1 peptides as specified above at a final concentration of 1 μg/ml. All peptides were synthesized at the LSU Protein Facility (Dr. Ted Gauthier). Phorbol myristic acid (50 ng/ml, Sigma) and ionomycin (1 μg/ml, Sigma) were used as positive controls for T cell activation. Negative controls had no antigen or mitogen stimulation. Brefeldin A (10 μg/ml, Sigma) was added to cultures after the first hour, followed by a 6-hour incubation. Following stimulation, cells were stained for cell surface markers with directly conjugated monoclonal antibodies to CD4 APC-Cy7 and CD8a pacific blue for 30 min at room temperature and washed with dPBS/BSA wash buffer. Cells were then fixed and permeabilized by using Cytofix/Cytoperm (BD Biosciences), washed twice in Perm Buffer (BD Biosciences), and stained with intracellular monoclonal antibodies against IFN-γ PE (XMG1.2, BD Biosciences) and TNF-α APC (MP6-XT22, BD Biosciences) were added to cells and incubated at room temperature for 30 min. Single color and isotype-matched control antibodies were used to confirm staining specificity. After washing, cells were resuspended in 1% paraformaldehyde in PBS and stored in the dark at 4°C. Data were acquired within 24 h of staining using a LSR II instrument (BD Immunocytometry System) and FACSDiva software (BD Immunocytometry System). For each sample, 50,000 events were collected by gating on CD4+ T cells. Data analysis was performed using FlowJo software. Cells were gated on singlets followed by CD4+ and CD8+ T cell subsets. Gated CD4+ and CD8+ T cells were further analyzed for its cytokine production. Positive cytokine responses were determined based on the percentage of cytokine responses obtained above background responses (unstimulated control) in each experiment. The cut off values were determined based on the cytokine responses obtained from mock-infected mice.

## Competing interests

The authors declare that they have no competing interests.

## Authors’ contributions

Most of the work was performed by AVI and BP who contributed equally to the work. VNC constructed the gK-null virus. JDW and BS helped validate the results and write the manuscript. KGK directed the entire work, interpreted and presented the data as the corresponding author. All authors read and approved the final manuscript.

## Supplementary Material

Additional file 1: Figure S1 Intracellular cytokine flow cytometry for IFN-γ responses from a representative vaccinated mouse. Splenocytes were left unstimulated (media control) or stimulated for 6 h with different gB and gD peptides. Cells were gated first on singlets followed by lymphocytes and CD4 and CD8 population. The percentages of IFN-γ positive cells are shown in each quadrant. Click here for file
