# Supplementary figures and images for: Single dose of Glycoprotein K (gK)-deleted HSV-1 live-attenuated virus protects mice against lethal vaginal challenge with HSV-1 and HSV-2 and induces lasting T cell memory immune responses
Source: Virol J. 2013 Oct 28;10:317. doi: 10.1186/1743-422X-10-317 (PMC3826548; doi:10.1186/1743-422X-10-317)

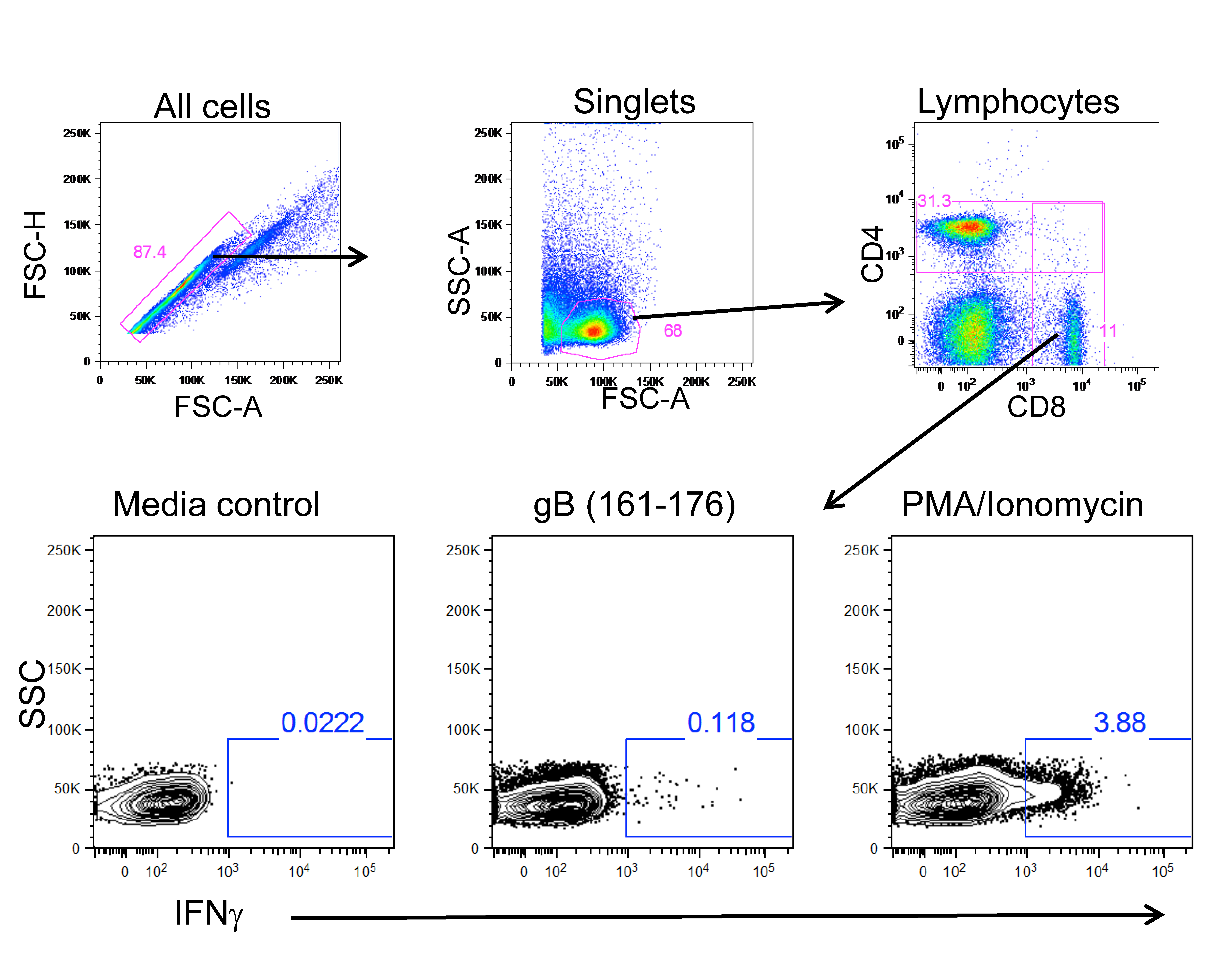

Supplement: Additional file 1: Figure S1 — Intracellular cytokine flow cytometry for IFN-γ responses from a representative vaccinated mouse. Splenocytes were left unstimulated (media control) or stimulated for 6 h with different gB and gD peptides. Cells were gated first on singlets followed by lymphocytes and CD4 and CD8 population. The percentages of IFN-γ positive cells are shown in each quadrant. [file 1743-422X-10-317-S1.tiff]
